# Supplementary material for: Commensal-derived Trehalose Monocorynomycolate Triggers γδ T Cell-driven Protective Ocular Barrier Immunity
Source: bioRxiv. 2025 Mar 18:2025.03.17.643820. Preprint. [Version 1] doi: 10.1101/2025.03.17.643820 (PMC11956969; doi:10.1101/2025.03.17.643820)
Supplement: 1 [file NIHPP2025.03.17.643820V1-supplement-1.pdf]

bioRxiv preprint doi: https://doi.org/10.1101/2025.03.17.643820; this version posted March 18, 2025. The copyright holder for this preprint (which was not certified by peer review) is the author/funder. This article is a US Government work. It is not subject to copyright under 17 USC 105 and is also made available for use under a CC0 license.

Supplementary Figures

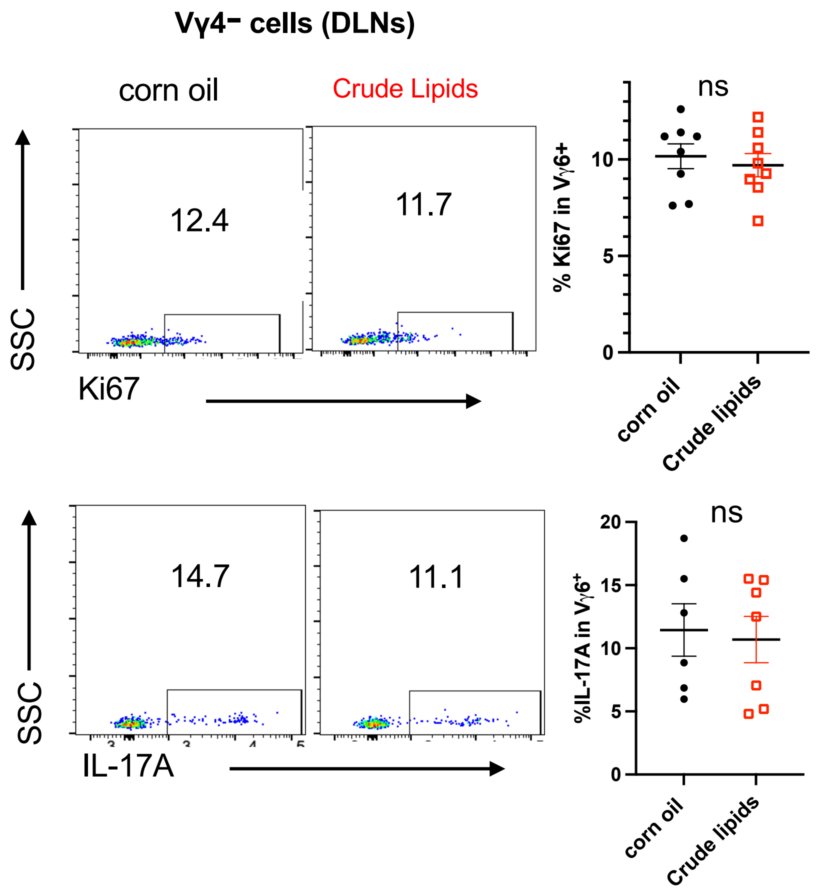

Figure S1. Lipid components of C. mast do not elicit IL-17 responses in Vγ4-negative cells (related to Figure 1)

Representative flow cytometry plots and dot plots showing the percentage of Ki67+ and IL-17A+ in Vγ4- cells of DLNs from mice described in Figure 1C. Each dot represents one animal. Bars represent mean ± SEM. *p<0.05, **p<0.01, ***p<0.001. Statistical significance was determined by the Mann-Whitney test.

bioRxiv preprint doi: https://doi.org/10.1101/2025.03.17.643820; this version posted March 18, 2025. The copyright holder for this preprint (which was not certified by peer review) is the author/funder. This article is a US Government work. It is not subject to copyright under 17 USC 105 and is also made available for use under a CC0 license.

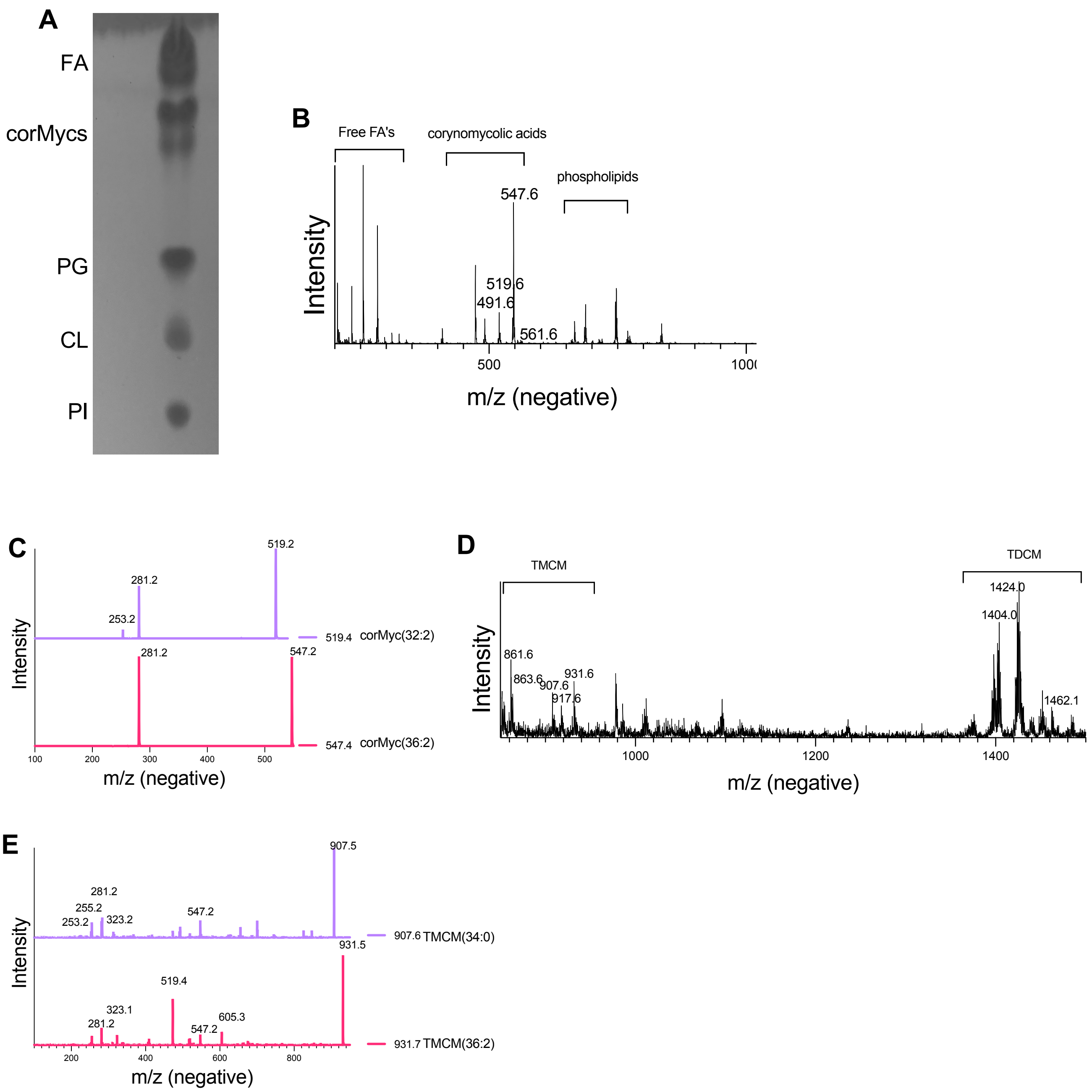

Figure S2. Identification and characterization of lipid classes in *C. mast* crude lipid extract (related to Figure 2)

(**A**) Crude lipids of *C. mast* were analyzed by thin layer chromatography(TLC).

(B) Full mass spectra under negative ionization conditions of crude lipid preparation from WT *C. mast*. 200-1100 m/z regions were populated by free fatty acids, corynomycolic acids(corMycs), and phospholipids(PG,CL,PI). Within the corynomycolic acid region, major peaks are anticipated to be corMyc(C32:2) at -519.6 (M-), corMyc(C36:2) at -547.6 (M-).

(C) Fragmentation spectra under negative polarity of the putative corMyc (32:2) at -519.6 and corMyc(C36:2) at -547.6 using rolling collision energy establishing these species as corMyc(a18:1/my18:1) under the -547.4 signal, indicated by the -281.2 product ion, and a mixture of corMyc (a16:1/my16:1) and corMyc (a18:1/my14:1) contributing to the -519.2 ion, indicated by the -253.2 and –281.2 product ions respectively.

(D) Zoomed spectra from the same injection as **(B)** for 850 -1500 m/z region of trehalose conjugated corynomycolates in WT *C.mast* crude lipid preparation. Putative acetate series (M+59-) for TMCM and TDCM species are labeled. The major isomeric contributors to each signal are anticipated to be TMCM (C34:0) at -907.5 (M+AcO-), and TMCM(C36:2) at -931.5 (M+AcO-), TDCM(72:4) at -1462.1 (M+AcO-), TDCM (68:4) at -1404.0 (M+AcO-).

(E) Fragmentation spectra of putative acetate series ions (M+59-) for TMCM(C34:0) at -907.5 (M+AcO-), and TMCM(C36:2) at -931.5 (M+AcO-) showing a distribution of species under each signal as evidenced by the series of fatty acid peaks in the 200-300 m/z fragment range.

bioRxiv preprint doi: https://doi.org/10.1101/2025.03.17.643820; this version posted March 18, 2025. The copyright holder for this preprint (which was not certified by peer review) is the author/funder. This article is a US Government work. It is not subject to copyright under 17 USC 105 and is also made available for use under a CC0 license.

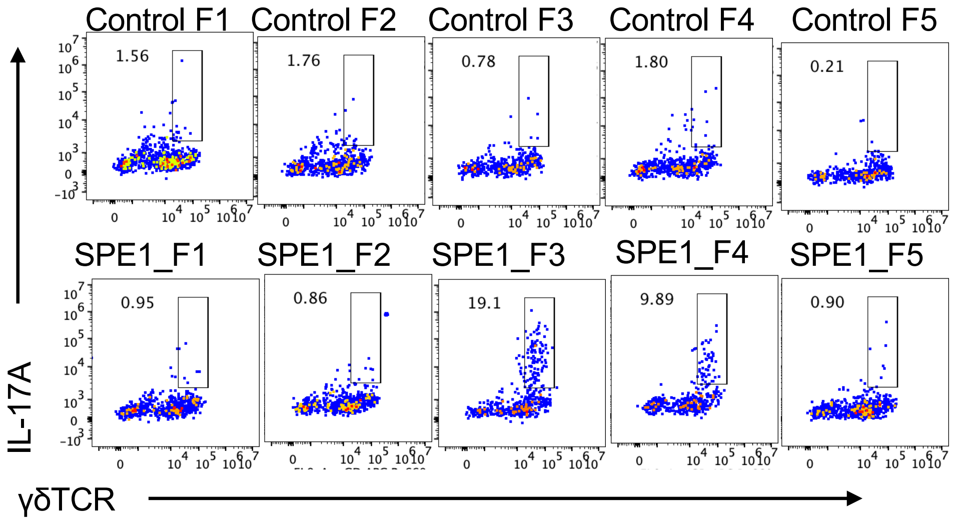

Figure S3. SPE1 fractions F3 and F4 contain il-17 stimulatory activity ***(*****related to Figure 2*****)***

Representative FACS plots showing the percentage of IL-17A in the co-cultures described in Figure 2D.

bioRxiv preprint doi: https://doi.org/10.1101/2025.03.17.643820; this version posted March 18, 2025. The copyright holder for this preprint (which was not certified by peer review) is the author/funder. This article is a US Government work. It is not subject to copyright under 17 USC 105 and is also made available for use under a CC0 license.

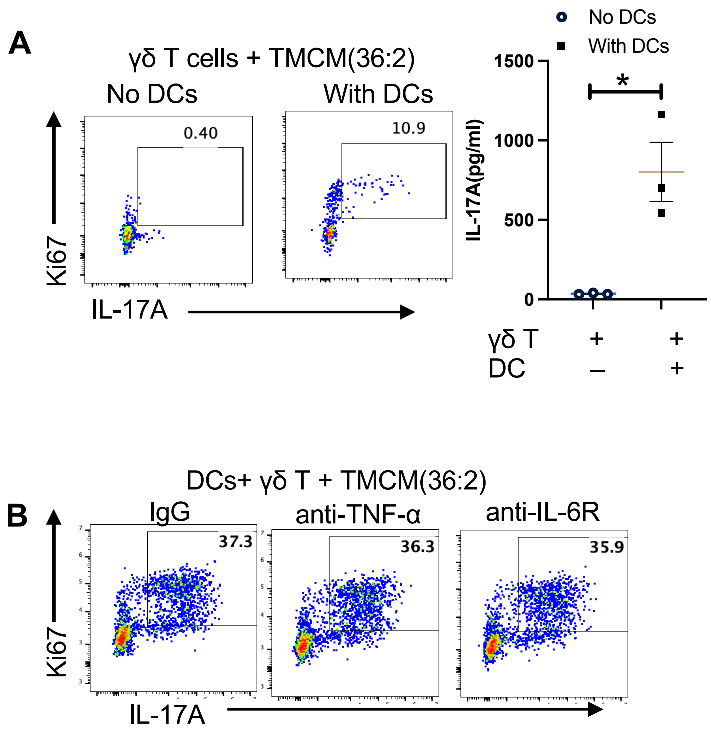

Figure S4. TNF-α and IL-6 affect TMCM-induced IL-17 responses when present together, but not individually *(*related to Figure 4*)*

(**A**) Sorted γδT cells alone or with CD11c+ DCs in the presence of TMCM(36:2) for 3 days. Representative flow cytometry plot showing the percentage of Ki67+IL-17A+ on gated γδT cells, and levels of IL-17A in the culture supernatant are shown in dot plot. Each dot represents one experiment. (**B**) Blockade antibodies targeting anti-TNF-a or anti-IL-6R were added in cocultures with sorted γδT cells with CD11c+ DCs in the presence of TMCM(36:2) for 2 days. Representative flow cytometry plot showing the percentage of Ki67+IL-17A+ on gated γδT cells. Representative data of at least 2 independent experiments.

Bars represent mean ± SEM. *p<0.05, **p<0.01, ***p<0.001. Statistical significance was determined by the Mann-Whitney test (A).

bioRxiv preprint doi: https://doi.org/10.1101/2025.03.17.643820; this version posted March 18, 2025. The copyright holder for this preprint (which was not certified by peer review) is the author/funder. This article is a US Government work. It is not subject to copyright under 17 USC 105 and is also made available for use under a CC0 license.

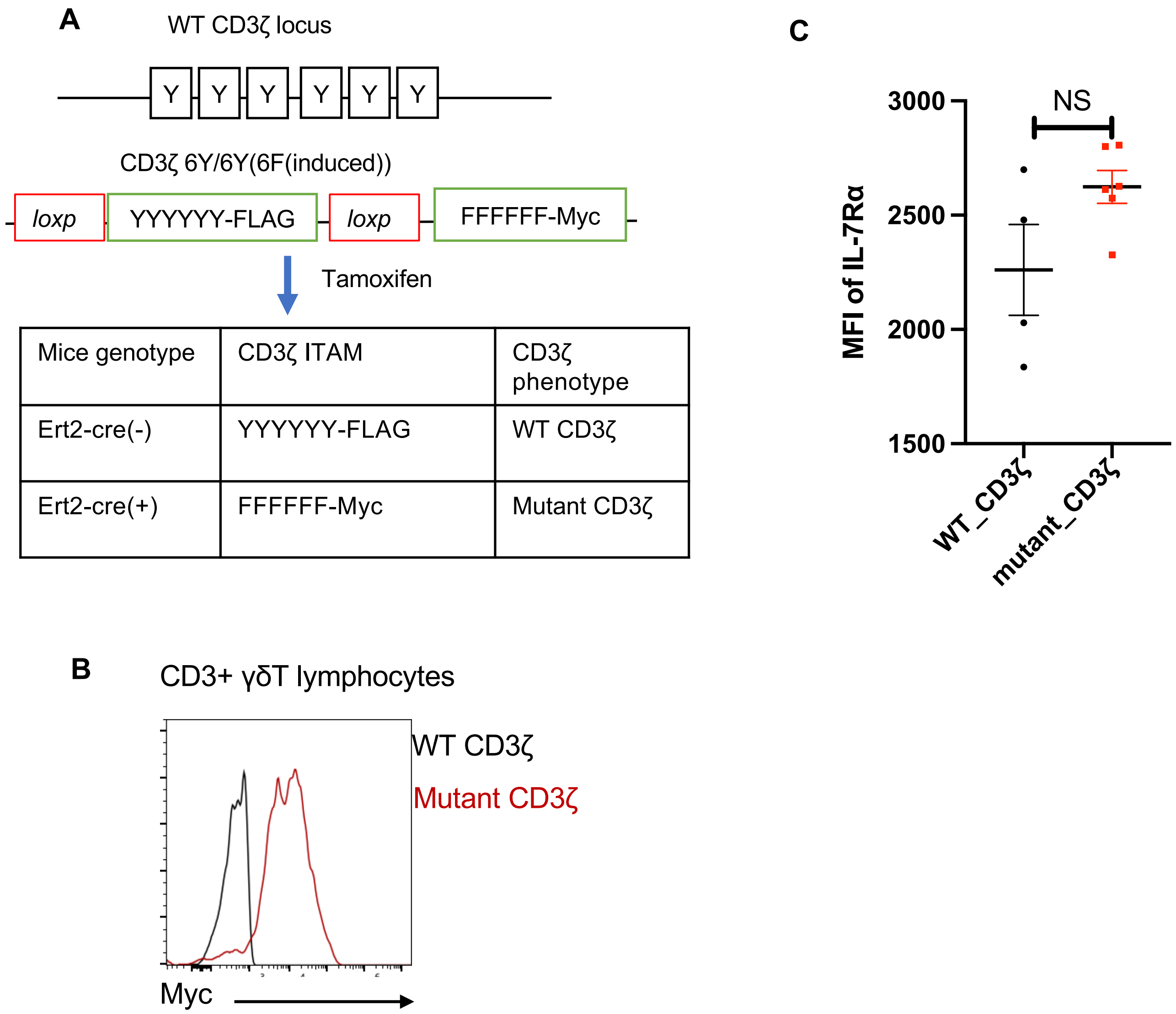

Figure S5. γδ TCR signaling does not affect IL-7R expression *(*related to Figure 5*)*

(**A**) Schematic representation of CD3ζ ITAM motif in Ert2-cre(-) and Ert2-cre(+) CD3ζ 6Y/6Y(6F(induced)) mice before and after tamoxifen induced modification. The tyrosines (Y) in the wild-type ITAM motif were replaced by phenylalanines(F) in mutant CD3ζ, impairing TCR signaling.
(**B**)The efficiency of mutant CD3 induction shown as Myc tag expression after tamoxifen treatment. Lymphocytes from WT and mutated CD3ζ mice stained with anti-Myc.
(**C**) The expression of IL-7Rα on CD44+CD27- γδ T cells from WT and mutated CD3ζ mice 8 days after tamoxifen treatment*.* Each dot represents an individual mouse.
The results were representative of at least 2 independent experiments.
Bars represent mean ± SEM. *p<0.05, **p<0.01, ***p<0.001.Statistical significance was determined by Mann-Whitney test (C).

bioRxiv preprint doi: https://doi.org/10.1101/2025.03.17.643820; this version posted March 18, 2025. The copyright holder for this preprint (which was not certified by peer review) is the author/funder. This article is a US Government work. It is not subject to copyright under 17 USC 105 and is also made available for use under a CC0 license.

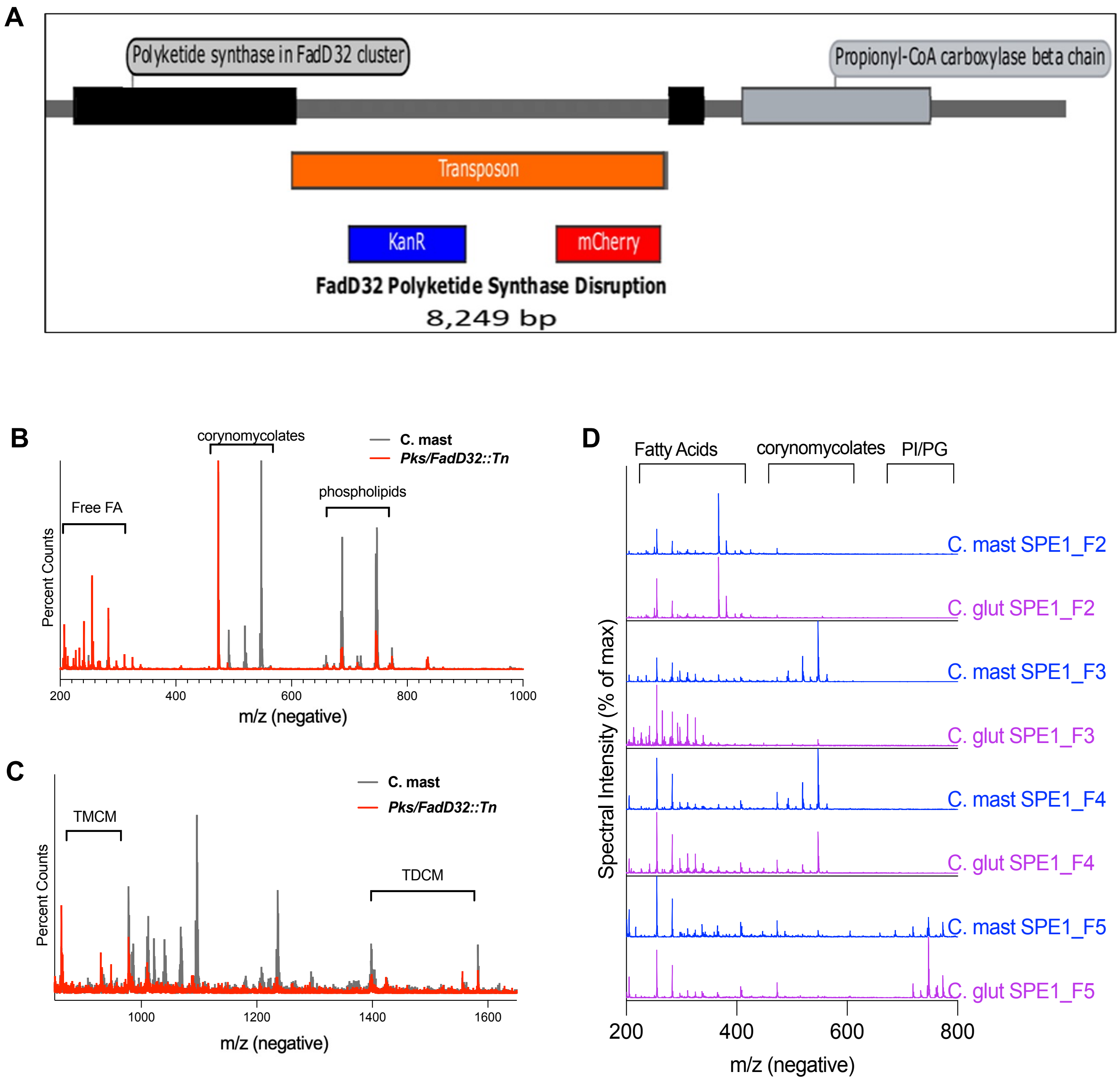

Figure S6. ***C. mast Pks/FadD32::Tn*** **lacks corynomycolates expressed by WT** ***C. mast*** **and** ***C. glut*** ***(*****related to Figure 6*****)***

(A) The whole genome of *Pks/FadD32::Tn* was sequenced, and the transposon (Tn) was inserted in FadD32 cluster polyketide synthase.
(B-C) Full mass spectra under negative ionization conditions of crude lipid preparation from WT *C. mast Pks/FadD32::Tn* mutant *C. mast*. 200-1000 m/z regions were populated by free fatty acids, corynomycolates (corMycs), and phospholipids (**B**). Zoomed spectra for 850 -1500 m/z region displayed trehalose conjugated corynomycolates in WT and *Pks/FadD32::Tn* mutant *C. mast* crude lipid preparation **(C)**.
(D) Lipid fractions from *C. mast* and *C. glut* lipidome were examined by liquid chromatography-mass spectrometry (LC-MS). F1 showed a redundant pattern to F2 and is not depicted.

bioRxiv preprint doi: https://doi.org/10.1101/2025.03.17.643820; this version posted March 18, 2025. The copyright holder for this preprint (which was not certified by peer review) is the author/funder. This article is a US Government work. It is not subject to copyright under 17 USC 105 and is also made available for use under a CC0 license.

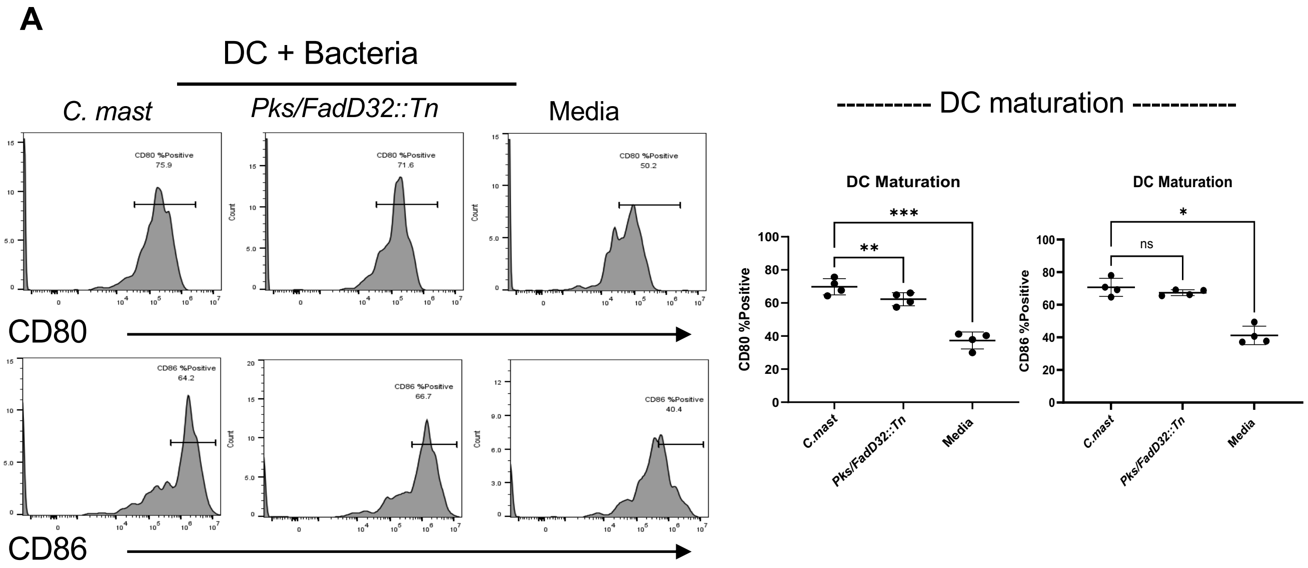

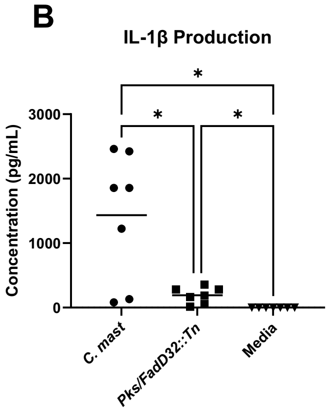

Figure S7. ***Pks/FadD32::Tn*** **mutant** ***of C. mast*** **induces a reduced DC IL-1β response (related to Figure 6)**

(A-B) $5x10^4$ BMDCs were co-cocultured with $1x10^5$ CFU of either WT *C. mast* or *Pks/FadD32::Tn*. After incubating for 48 hours, BMDCs were assessed for DC maturation through expression of CD80 and CD86 (**A**), and supernatants were analyzed for IL-1β by ELISA (**B**). In all graphs, bars represent mean ± SEM. Symbols represent individual experiments. Significance was determined using paired Brown-Forsythe and Welch ANOVA (A) or one-way ANOVA (B) (*p= 0.0049, ***p= 0.0001).

bioRxiv preprint doi: https://doi.org/10.1101/2025.03.17.643820; this version posted March 18, 2025. The copyright holder for this preprint (which was not certified by peer review) is the author/funder. This article is a US Government work. It is not subject to copyright under 17 USC 105 and is also made available for use under a CC0 license.

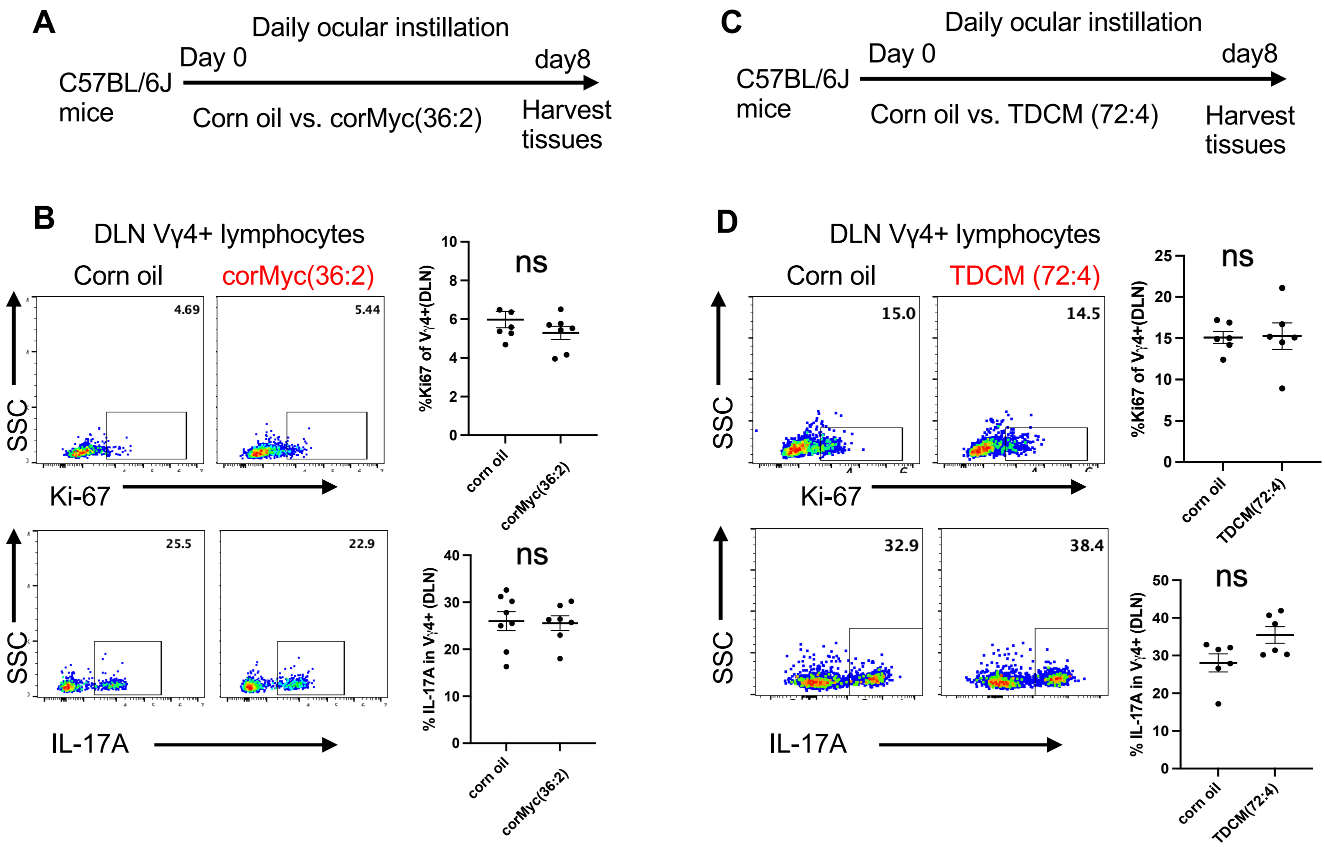

Figure S8. corMyc (36:2) and TDCM (72:4) do not elicit ocular IL-17 responses in healthy mice *(related to Figure 7)*

(**A-B**) Experimental design to assess ocular immune responses elicited by synthetic corMyc (36:2). (**A**) Corn oil (vehicle control) or 25 µg synthetic corMyc (36:2) was instilled on the ocular surface of C57BL/6J mice daily for 7 days. (**B**) Representative flow cytometric analysis and dot plots show the percentage of Ki67+ or IL-17A+ Vγ4 cells in the eye-draining cervical LNs (DLNs). Each dot represents one animal.
(C-D) Experimental design to assess ocular immune responses elicited by synthetic TDCM(72:4). (**C**) Corn oil (vehicle control) or 25 µg synthetic TDCM(72:4) was instilled on the ocular surface of C57BL/6J mice daily for 7 days. (**D**) Representative flow cytometric analysis and dot plots show the percentage of Ki67+ or IL-17A+ Vγ4 cells in the eye-draining cervical LNs (DLNs). Each dot represents one animal.
The results were representative of at least 2 independent experiments.
Bars represent mean ± SEM with *p<0.05, **p<0.01, ***p<0.001. Statistical significance was determined by Mann-Whitney test. ns, not significant (**B,D).**
